# Editorial: Application of rehabilomics in surgical conditions

**DOI:** 10.3389/fsurg.2024.1493770

**Published:** 2024-10-15

**Authors:** Huaide Qiu, Raquel Alarcon Rodriguez, Yuxuan Song

**Affiliations:** ^1^ Faculty of Rehabilitation Science, Nanjing Normal University of Special Education, Nanjing, China; ^2^ Department of Nursing, Physiotherapy and Medicine, Faculty of Health Sciences, University of Almería, Almería, Spain; ^3^ Department of Urology, Peking University People’s Hospital, Beijing, China; ^4^ The Institute of Applied Lithotripsy Technology, Peking University, Beijing, China

**Keywords:** rehabilomics, surgery, biomarkers, functioning, multi-omic analysis

**Editorial on the Research Topic**
Application of rehabilomics in surgical conditions

Rehabilomics stands out as a holistic research framework that extends beyond the conventional boundaries of rehabilitation (1). It embraces a wide range of omics data, including genomics, proteomics, and metabolomics, to unravel the complex biological underpinnings of individual responses to rehabilitation interventions (1). This multi-dimensional approach allows for a more nuanced understanding of a patient's condition, moving away from a one-size-fits-all model towards a tailored treatment plan that is finely attuned to the patient's unique biological profile.

**Figure 1:**
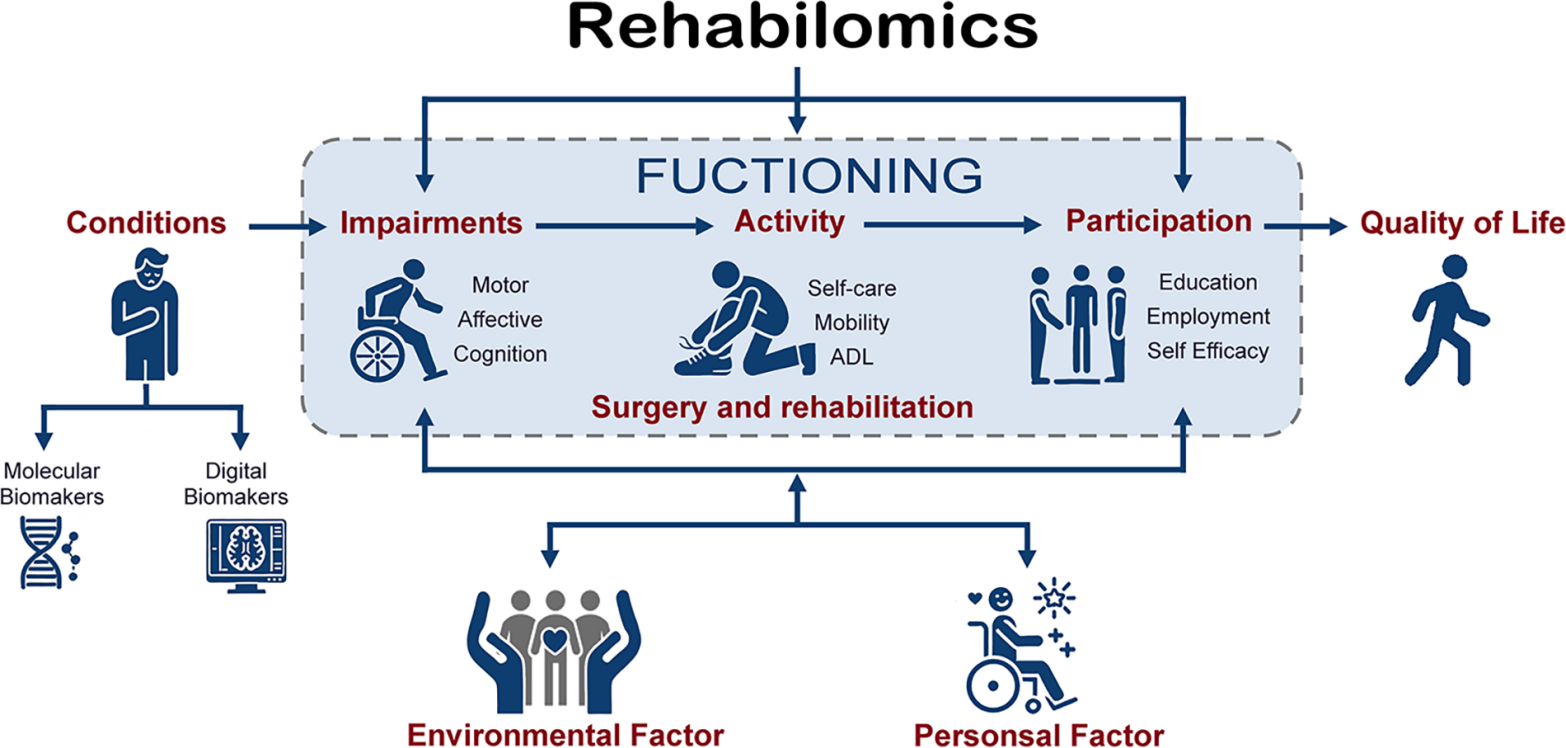
Framework of rehabilomics in surgical conditions.

In the realm of surgical conditions, the advent of Rehabilomics marks a paradigm shift towards a more personalized and precise approach to patient care. Figure 1 represents a conceptual framework for understanding the relationship between various factors affecting an individual's health and quality of life in the context of “Rehabilomics”. This special topic aims to delve into the burgeoning field of Rehabilomics, which integrates omics technologies with rehabilitation practices to enhance functional evaluation (2), outcome prediction (3), and personalized treatment strategies (4).

Yu et al. presents a comparative analysis of computed tomography (CT)-guided radiofrequency ablation (RA) and alcohol ablation (AA) for treating primary hyperhidrosis, focusing on the head and palms. The retrospective analysis involved 54 patients and divided them into RA (30 cases) and AA (24 cases) groups. The study evaluated treatment effectiveness, patient satisfaction, quality of life, safety, and the number of CT scans required for each procedure. Key findings indicate that both RA and AA significantly improved hyperhidrosis symptoms, with 80% satisfaction in the RA group and 79.2% in the AA group, showing no significant difference in treatment efficacy or complications (all *P* > 0.05). However, RA required fewer CT scans (4.60 ± 0.56) compared to AA (6.08 ± 0.28), which was statistically significant (*P* < 0.05). Complications were minimal and similar between groups, including pneumothorax, hemothorax, and compensatory hyperhidrosis. The study concludes that both RA and AA are effective minimally invasive treatments for hyperhidrosis, with comparable safety and efficacy profiles. However, RA is associated with a lower number of CT scans, suggesting a potential advantage in procedural efficiency.

Dong et al. details the surgical intervention and rehabilitation process for a rare instance of bilateral spontaneous quadriceps tendon rupture. The patient, a 44-year-old woman, experienced simultaneous rupture of both quadriceps tendons after a fall, leading to acute knee pain and limited mobility. Diagnostic confirmation via physical examination and magnetic resonance imaging scans was followed by surgical repair, which included a modified Mason-Allen suture technique and wire looping to reduce tendon tension. Postoperatively, a comprehensive rehabilitation program was initiated, focusing on pain management, muscle strength recovery, and knee joint mobility enhancement. The patient demonstrated significant improvements in pain reduction, as measured by the Visual Analogue Scale, and in muscle strength, achieving a full recovery by the third month post-surgery. Knee range of motion also progressively improved, allowing the patient to regain functional capabilities necessary for daily activities. The study emphasizes the importance of early diagnosis, precise surgical treatment, and tailored rehabilitation for optimal recovery in such cases. It also highlights the potential role of genetic factors and calcium-phosphorus metabolism in the etiology of spontaneous quadriceps tendon ruptures, suggesting avenues for future research. The case underscores the multidisciplinary approach necessary for managing this rare condition, integrating orthopedic surgery with rehabilitation and considering underlying systemic health.

The narrative review by Youssef et al. explores the digitalization in orthopaedics, highlighting the impact of technological advancements such as the Internet of Things, artificial intelligence (AI), and sensors on patient care, research, and logistics. The coronavirus disease (COVID-19) pandemic has accelerated the adoption of telemedicine, with digital tools facilitating remote patient monitoring, online consultations, and data-driven healthcare. The use of AI in medical imaging, 3D modeling, and robotic-assisted surgery enhances precision and efficiency. However, challenges remain, including ensuring data privacy, addressing technical limitations, and navigating regulatory frameworks. The review underscores the potential for digitalization to revolutionize orthopaedics through personalized patient care and interdisciplinary collaboration.

Li et al. utilizes a machine learning approach to predict the 90-day prognosis of patients with hypertensive intracerebral hemorrhage following stereotactic hematoma removal. The research retrospectively analyzes 432 patients, extracting clinical and radiomic data to develop predictive models. Key findings indicate that a nomogram integrating clinical and radiomic features achieves superior predictive accuracy compared to models relying on either data type alone, with area under the curves of 0.987 and 0.932 in training and test sets, respectively. This highlights the potential of multimodal data fusion in enhancing prognostic assessment, offering a promising tool for clinicians to individualize risk evaluation and treatment strategies for hypertensive intracerebral hemorrhage patients post-stereotactic surgery.

In summary, as Rehabilomics continues to evolve, its potential to significantly impact public health is becoming increasingly evident. Rehabilomics is at the forefront of revolutionizing post-surgical recovery by offering insights into the mechanisms of rehabilitation therapies. By examining the interplay between biological factors and rehabilitation strategies, this field paves the way for uncovering the intricate mechanisms that govern recovery and functional restoration. This knowledge is transformative, as it not only informs the development of novel therapeutic approaches but also enhances the efficacy of existing rehabilitation. Its application in surgical conditions is a testament to the power of integrating advanced omics research with clinical practice, offering a future where rehabilitation is not just a process of recovery but a journey of personalized healing and optimized outcomes.
